# Enhancing antimicrobial stewardship using a retrieval-augmented generation large language model for infectious disease management

**DOI:** 10.31744/einstein_journal/2026AO2175

**Published:** 2026-07-22

**Authors:** Hugo Morales, Cristian Rocha, Luan Matheus Trindade Dalmazo, Lucas de Azevedo Takara, Mateus Cichelero, Bruno Guerra, Jessica Andrade-Silva, Paula Tuma, Moacyr Silva

**Affiliations:** 1 Munai São Paulo SP Brazil Munai, São Paulo, SP, Brazil.; 2 Pontifícia Universidade Católica Curitiba PR Brazil Pontifícia Universidade Católica, Curitiba, PR, Brazil.; 3 Universidade Federal do Paraná Curitiba PR Brazil Universidade Federal do Paraná, Curitiba, PR, Brazil.; 4 Hospital Israelita Albert Einstein São Paulo SP Brazil Hospital Israelita Albert Einstein, São Paulo, SP, Brazil.

**Keywords:** Large language models, Artificial Intelligence, Antimicrobial stewardship, Antimicrobial resistance, Information storage and retrieval, Communicable diseases

## Abstract

**Objective:**

This study developed and evaluateed a retrieval-augmented generation (RAG)-based large language model system designed to standardize and improve antimicrobial treatment protocol adherence, leveraging the Antimicrobial Treatment Guide from the *Hospital Israelita Albert Einstein* in São Paulo, Brazil.

**Methods:**

Four modules were used to compose the search system, namely document processing, query processing, large language model, and prompt management. Protocol documents were manually segmented into manageable text chunks, converted into vectors, and stored in a vector database to enable efficient retrieval using the RAG methodology. This approach allows the model to generate responses based on external data without additional training. The evaluation included quantitative metrics, such as the Jaccard index for retrieval accuracy, Public Health System (SUS - *Sistema Único de Saúde*) usability scale, and behavior change questionnaire to assess user perception and impact on clinical decision-making.

**Results:**

The system achieved an SUS score of 82, indicating excellent usability. The system further correctly retrieved the appropriate antimicrobial protocol in 83% of cases. These results suggest effective alignment between system responses and institutional clinical guidelines.

**Conclusion:**

The RAG-based system demonstrated high usability and reliable retrieval performance, supporting standardized antimicrobial therapy aligned with local guidelines. This approach has the potential to bridge the gap between clinical practice and protocol adherence, enhance diagnostic accuracy and treatment specificity, and potentially reduce the risk of antibiotic resistance while improving patient outcomes.

## Highlights

An large language models and RAG-based system for antimicrobial treatment guideline retrieval.

System evaluation with an emphasis on usability and trust.

Discussion of potential tool applications in healthcare.

## In Brief

Antimicrobial stewardship is important for proper and accurate infection treatment as well as preventing resistance. This study introduces a large language model-based Antimicrobial Treatment Guide search tool at the *Hospital Israelita Albert Einstein*.

## Graphical abstract

**Figure:**
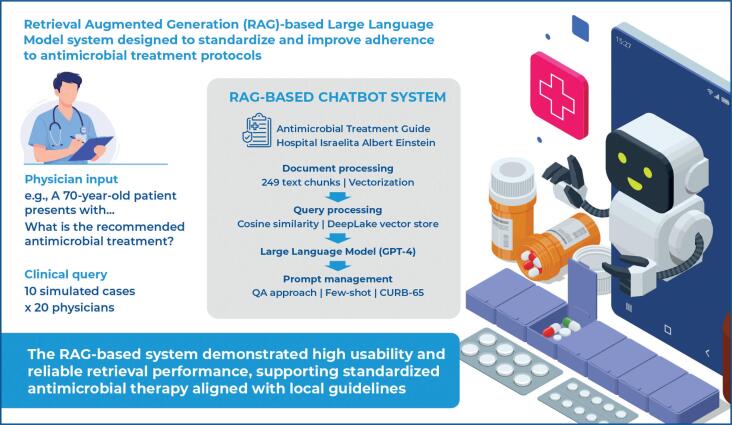

## INTRODUCTION

Timely and efficient infectious disease diagnosis and management within hospital environments are crucial for enhancing patient clinical outcomes, reducing selective pressure, and subsequently lowering antibiotic resistance risk. However, hospital guideline adherence for rapid and precise treatment is often problematic for clinicians. Case complexity, patient symptom variability, and high-pressure environment contribute to suboptimal guideline compliance, which can lead to inferior treatment outcomes and an increased risk of antibiotic resistance.

Integrating large language models (LLMs) in healthcare settings has shown potential regarding substantially enhancing clinical decision-making and patient management.^(1)^ across various departments. Using extensive datasets and advanced algorithms, these models can provide healthcare professionals with real-time, contextually relevant information that closely aligns with hospital guidelines to improve treatment accuracy and speed.

Despite LLM advancements in healthcare, notable limitations that constrain their full potential exist. For instance, integrating user experience feedback into the model learning and adaptation processes.^(2)^ Large language models often lack mechanisms to effectively incorporate direct input from clinicians who interact with these systems daily, which can result in tools that are unintuitive or fail to align with the practical needs of healthcare providers.

Additionally, LLMs are typically trained on broad, heterogeneous datasets that may not accurately reflect the specific epidemiology, prescribing patterns, or institutional practices captured in the specific guidelines of a hospital. Large language models models can also provide inaccurate or incomplete recommendations.^(3)^ Moreover, maintaining such models up to date with evolving evidence and local protocols requires ongoing maintenance and retraining, which can be resource-intensive and difficult to sustain in a fast-paced clinical environment.

**Figure 1:**
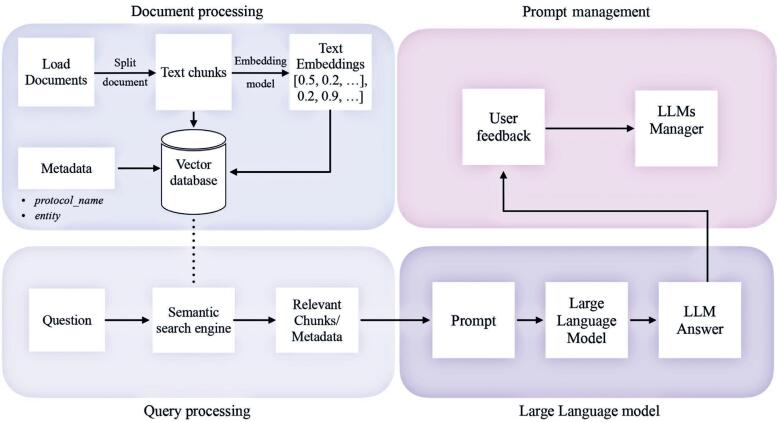
Fluxogram of the retrieval augmented generation pipeline

This study proposes a method to enhance antimicrobial treatment protocols across hospitals by integrating a retrieval-augmented generation (RAG) into an LLM. This approach leverages the Antimicrobial Treatment Guide from the *Hospital Israelita Albert Einstein*^(4)^ to standardize antimicrobial therapy according to the hospital. The model is based on the RAG methodology, which utilizes external data sources to generate responses without the need for extensive training. The system comprises four main modules: document processing, query processing, LLM, and prompt management (Figure 1). These modules encompass file analysis, query handling, the LLM itself, and user interaction.

Evaluating the RAG method for hospital antimicrobial treatment protocols uses several metrics to assess performance and usability. Quantitative performance is measured using the Jaccard index, which calculates the similarity between the predicted and actual treatments. System usability is assessed using the Public Health System (SUS - *Sistema Único de Saúde*) usability scale, and a behavior change questionnaire, which evaluates aspects such as confidence, frequency of use, and decision-making assistance. Collectively, a comprehensive overview of the system effectiveness and user experience is established in a hospital setting. This study focuses primarily on detailing the practical application of a RAG-based LLM to support antimicrobial stewardship within a clinical workflow, followed by describing the architecture used.

The main contributions of this study are developing an RAG-enhanced LLM tool to support antimicrobial protocols, integrating hospital guidelines to improve treatment specificity, and ensuring alignment with clinical practices and patient needs as well as evaluating 10 simulated medical cases from the *Hospital Israelita Albert Einstein* to validate system performance in real-world scenarios.

The remainder of this paper is organized as follows: Related works are covered in Section 2, the adopted methodology and materials are detailed in Section 3, and result analysis and discussion are presented in Section 4. Finally, the paper is concluded in Section 5 and future research directions are discussed.

### Related work

This section discusses some applications of large language models in healthcare and their implications. This technology has garnered increasing attention. Literature reviews, such as those published in Future Internet, demonstrate the extensive applications of generative Artificial Intelligence (AI) in various medical and healthcare fields.^(5)^

Several pivotal studies have demonstrated the profound impact of AI models across diverse medical fields. Esteva et al. explored how AI optimizes the management and accessibility of electronic health records, thereby enhancing patient care quality.^(6)^ Automation streamlines healthcare workflows, allowing medical professionals to dedicate additional time to direct patient interactions, thereby considerably improving service delivery and operational efficiency.

The use of AI in decision-making processes within clinical environments, including diagnostics and personalized treatment strategies, has also been evaluated.^(7)^ Previous studies focused on technologies that enable a nuanced interpretation of complex medical datasets, facilitating timely interventions that are crucial for effective patient care.

However, using such advanced technologies in healthcare also introduces substantial ethical challenges. Char et al. discussed the critical considerations required to ensure the ethical implementation of AI in healthcare contexts.^(8)^ Their research underscores the necessity of safeguarding patient privacy, mitigating biases in AI algorithms, and enhancing AI-driven decision transparency.

## OBJECTIVE

This study details RAG system development and evaluation powered by large language model, designed to facilitate and standardize adherence to the clinical protocols outlined in the Antimicrobial Treatment Guide of the *Hospital Israelita Albert Einstein*.

## METHODS

This section presents the developed RAG-based clinical decision support tool, its integration with hospital antimicrobial guidelines, and the methodology used to evaluate its practical use within clinical workflows. Implementation details, including hyperparameter tuning and experimental settings, are provided to clarify how the system operates and how its performance was assessed. The study was approved by the local Ethics Committee of the *Hospital Israelita Albert Einstein* (CAAE: 65886322.4.0000.0071; # 6.019.786).

### System overview

The RAG methodology^(9)^ was adopted as the core of the tool because of its capacity to generate responses based on external data sources without the need for fine-tuning. Figure 1 presents the overall architecture, which is organized into four main modules: document processing, query processing, LLM, and prompt management.

### Data collection and processing

The external data pertains to the Antimicrobial Treatment Guide deployed at the *Hospital Israelita Albert Einstein* (see Section "Availability of data and materials" for access to the guides in PDF), focusing on medical practices intended to standardize and rationalize the use of antimicrobial therapy in hospitalized adult patients. This guide is crucial for ensuring appropriate infection management and aims to reduce the risk of antimicrobial resistance and enhance patient care outcomes.

The document encompasses specific guidelines for managing prevalent infections encountered in the hospital: community-acquired pneumonia, skin infections (erysipelas and cellulitis), urinary tract infection, community-acquired meningitis, gastroenterocolitis, and intensive care unit (ICU) antimicrobial treatment guide.

The infections and their corresponding treatment guidelines are organized in both tabular and textual formats within the document. The guidelines include several critical parameters, such as the causative agent or diagnosis, primary antibiotic therapy, alternative therapeutic options, management strategies for patients with β-lactam allergies, and recommended treatment durations. Table 1 presents the guidelines for the protocol addressing community-acquired meningitis.

**Table 1:** Community meningitis antimicrobial treatment guide in the adult intensive care unit

Agent	Antibiotic therapy	Alternative	Allergic to β-lactam	Treatment duration/days
Empirical	Ceftriaxone 2g IV every 12h	Meropenem 2g IV every 8 h	X	14
*Haemophilus influenzae*	Ceftriaxone 2g IV every 12h	Cefepime 2g IV every 8 h	Meropenem 2g IV every 8 h	10
*Neisseria meningitidis*	Ceftriaxone 2g IV every 12h	Meropenem 2g IV every 8 h	Meropenem 2g IV every 8 h	7
*Streptococcus pneumoniae*	Ceftriaxone 2g IV every 12h (Ceftriaxone MIC <1.0μg/mL)	Vancomycin (protocol) + Ceftriaxone 2 g IV every 12 h	Meropenem 2g IV every 8 h	14
*S. pneumoniae*	Vancomycin (protocol) + Ceftriaxone 2g IV every 12h	Ceftriaxone MIC >1.0μg/mL	Meropenem 2g IV every 8 h	14
*Enterobacteriaceae*	Ceftriaxone 2g IV every 12h	Meropenem 2g IV every 8 h	Meropenem 2g IV every 8 h	14–21
*Listeria monocytogenes*	Ampicillin 2g IV every 4h	Sulfamethoxazole/ trimethoprim 20mg/kg/day 6 h	Meropenem 2g IV every 8 h	21
*S. agalactiae*	Ampicillin 2g IV every 4h	Ceftriaxone 2g IV every 12 h	Meropenem 2g IV every 8 h or Vancomycin (protocol)	14
*Staphylococcus aureus* (oxacillin-sensitive)	Oxacillin 2g IV every 4h	Linezolid 600mg IV every 12 h	Daptomycin 12mg IV once daily	21
*Staphylococcus aureus* (oxacillin-resistant)	Vancomycin (protocol)	Linezolid 600mg IV every 12 h	Daptomycin 12mg IV once daily or Ceftaroline 600mg IV every 12 h	21

IV: intravenous MIC: minimum inhibitory concentration.

The *Agent* column identifies the etiological agent, guiding clinicians in selecting effective targeted antimicrobial therapy; *Antibiotic Therapy* column details primary antibiotics, including dosages and frequencies, for each condition; *Alternative* column provides secondary options when primary treatments are contraindicated due to specific patient conditions; *Allergic to β-lactam* column lists alternatives for those allergic to β-lactam antibiotics; and *Treatment Duration* column specifies the duration necessary to treat the infection.

Moreover, the document encompasses general guidelines and metadata, such as bibliographic references, identification details, versioning, creation, and revision timestamps, as well as information about the reviewers and authors. This compilation enhances the scalability and robustness of the adopted antimicrobial therapies by supporting ongoing medical education and protocol review.

The document is subsequently partitioned into small text segments following the RAG methodology. Considering the limited number of protocols, segmentation occurs manually through meticulous curation for each of the five infections and general guidelines. Each segment is organized in a tabular format under the column chunk_of_text. In tabular data, such as in table 1, all columns are merged, representing the information as a single text chunk. For text-based data, such as bibliographic references, identification details, and versioning, all data are combined into a single chunk for each protocol.

A character-based text splitter from LangChain^(10)^ was applied to each row of the texts to produce chunks with a total size of 1000 characters. Each new line prompts the creation of a new chunk. Overall, 249 text chunks were generated: 27 for community-acquired pneumonia, 30 for skin infections: erysipelas and cellulitis, 44 for urinary tract infection, 15 for community-acquired meningitis, 55 for gastroenterocolitis, and 77 for other general guidelines of the Antimicrobial Treatment Guide.

The *text-embedding-ada-002* model of OpenAI^(11)^ was used to transform text chunks into vectors. Finally, the metadata, including the *protocol_name* and its *entity*, along with the original text chunk and its text embeddings, were appended into the vector database managed by ActiveLoop.^(12)^ The stored data format includes *text_chunk*, *metadata*, *embedding*, and *id*, where *text_chunk* is the original text, *metadata* contains the name of the protocol and entity that created these standards, and *id* represents the unique identifier of the text chunk.

### Query processing

The query processing module handles user queries by embedding the input using the same method used for the documents and retrieving the most relevant guideline chunks from the DeepLake vector store, which supports both semantic similarity and metadata-based search. By converting queries and guideline chunks into dense vector representations using the same embedding model, the system identifies conceptually similar content and surfaces the most relevant antimicrobial recommendations.

The similarity between vector embeddings was quantified using the cosine similarity metric, defined as shown in Equation 1.


$$\text{Cosine}\;\text{Similarity}\left(\vec{A},\vec{B}\right)=\frac{\vec{A}\cdot \vec{B}}{\left|\vec{A}\right|\left|\vec{B}\right|}$$

where vector A and vector B represent the embeddings of two text chunks, vector A · vector B is the dot product, and |vector A| and |vector B| are the magnitudes of the respective vectors. This metric efficiently evaluates the directional alignment of the vectors, reflecting the degree of semantic similarity between the query and stored text chunks.

In addition, VectorStore^(13)^ incorporated a metadata search functionality that filtered and retrieved based on structured data associated with the text chunks. The *protocol_name* and *entity* metadata elements served as the criteria for filtering and refining search results. These attributes enabled efficient querying and enhanced semantic search capabilities by providing a mechanism to narrow down results based on user-defined parameters.

### Large language models

large language models are widely used in the natural language processing field. These models are built using a transformer architecture, introduced in 2017,^(14)^ which establishes a new state-of-the-art in text classification and comprehension tasks through attention mechanisms.

This module is responsible for generating responses to user queries. The prompt receives relevant chunks and metadata similar to the query from the previous module and uses these as context to answer questions.

A question-and-answer (QA) approach-based prompt was developed to enhance user interaction and deliver objective, question-aligned responses. This method utilizes a few-shot learning technique, enabling the model to generalize from a minimal set of examples. The prompt is also designed to facilitate quick decision-making by incorporating the calculation of the confusion, urea, respiratory rate, blood pressure, and age ≥65 years score (CURB-65), which is performed by the prompt using the provided input and tailors the response to the severity level of the case in question.

The prompt is divided into five sections: Overview, Special Handling for Protocol Questions, Correct Response Path for Listing Protocols, Handling of Non-Clinical Interactions, CURB-65 Guidance, and General Principles. As the model was trained for use in the Brazilian context, all responses are generated in Portuguese. However, for this study, these responses were translated into English.

Initially, the chatbot was described in detail, outlining its behavior, objectives, and background to ensure a personalized and targeted approach within the clinical environment. Consequently, all its responses would draw on the knowledge of clinical science, medical mechanisms underlying health and disease, patient care, and therapeutic approaches of the model.

To allow for instant recognition of questions regarding the protocols wherein the model was trained, the chatbot delivers direct responses to queries such as, "Which protocols have you been trained in?" The model identifies these questions by looking for key patterns, such as "protocols," "trained," and "system."

In the case of direct questions regarding the list of protocols, the immediate response will be: "The protocols available in the system and that I have been trained in are: community-acquired pneumonia, guide to antimicrobial treatment in the adult ICU, erysipelas and cellulitis, acute gastroenterocolitis in adults, urinary tract infection, and meningitis."

For interactions that do not specifically address the clinical environment, such as small talk, the chatbot will provide concise responses. However, for cases related to the clinical setting, each response will include the protocol on which the details are based. If the list of protocols does not contain the necessary information to provide an answer, papers and websites will be considered and properly referenced by the tool. Additionally, the model is required to include the medication and duration of treatment in its response. If this information is unavailable for a given case, it will be explicitly stated.

For QA related to the CURB-65 score calculation, guidance will only be provided if at least two of the following data points are provided: confusion, urea, respiratory rate, blood pressure, or age ≥65 years. To optimize performance for responses associated with this calculation, the chatbot is supplied with example questions and the expected answers for such cases.

Key principles are outlined to ensure that the chatbot delivers well-refined responses. Each reply will clearly cite the source of the information without repeating the user's question. Guidance related to protocols will be provided directly, based on the list of referenced protocols, and supported by external sources, such as academic papers. Finally, the chatbot is prohibited from recommending medications or providing guidance that are not covered within the listed protocols.

Once the prompt context is loaded and ready, the GPT-4^(15)^ model generates the corresponding answer. GPT-4 analyzes the input text**,** comprising user queries and defined prompt variables**,** to identify the underlying intent and relevant details. Therefore, GPT-4 synthesizes information and generates a response that answers the user's question and adheres to the specific guidelines set in the prompt, such as medical protocol adherence and response structure.

### Prompt management and evaluation

ChainLit^(16)^ and LiteralAI^(17)^ were used to implement the prompt management and monitoring module for the LLM application. This module logs user interactions and model outputs, enabling the tracking of task completion, response latency, and key quality indicators, such as alignment with institutional guidelines and suspected hallucinations. The collected traces support real-time dashboards and analyses that summarize response accuracy and user engagement. In this study, these tools were primarily used to support the systematic evaluation of the system's behavior and inform iterative refinements of the application.

### Evaluation metrics

This section outlines the evaluation process, which is divided into two phases: the first focuses on performance, measured by the number of correct protocols retrieved, and the second analyzes usability to determine whether the system provides reasonable interactions and accurate answers.

### Performance metrics

The Jaccard index is a common metric used to evaluate performance and is defined as in Equation 2.


$$\text{Jaccard}\;\text{index}=\frac{TP}{TP+FP+FN}$$

where the index is calculated by dividing the true positives (TP) by the exact sum of the previous terms with the sum of the false positive (FP) and false negative (FN).

### System usability scale and behavioral score

To assess tool usability and behavioral changes that its use could potentially cause among physicians, two questionnaires were administered: the SUS^(18)^ and a custom behavior change questionnaire. The custom behavior change questionnaire aimed to evaluate three main categories: confidence, frequency of use, and assistance in decision-making. The results were evaluated using the standard SUS calculator. Other outcomes were summarized based on the mean or percentage of cases in each category.

## RESULTS

### System usability results

The project involved 20 physicians, with data on sex and age available for 16 (N). The sex distribution was 37.5% female and 62.5% male (Table 2). The median age of the physicians was 43 years (range, 31–73 years). The median duration of professional experience for 15 physicians was 19 years (range, 7–50). The median SUS score was 82 points. This value, according to the questionnaire interpretation manuals, indicates a category of grade "Excellent" in 31.3% (5) of cases and "Good" in 56.3% (9). The positive categories marked as excellent and good were observed in most physicians (87.6%).

**Table 2:** Epidemiological profile of the physicians who tested the tool

Variable		n	Statistics
Age		16	43 (range 31–73)
Sex – n (%)		
	Male		10 (62.5)
	Female		6 (37.5)
Years since graduation		16	19 (range 7–50)
SUS Categorial – n (%)		
	Excellent		5 (31.3)
	Good		9 (56.3)
	Poor		2 (12.5)

**Figure 2:**
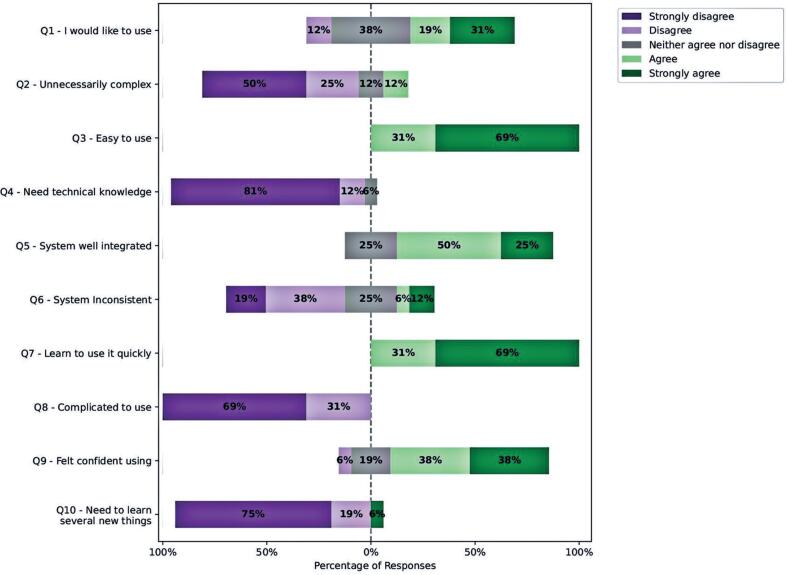
System usability scale

Each SUS question elucidated the main strengths or flaws of the chatbot (Figure 2). The paired question set of "I found the system easy to use"/"I found the system complicated to use," along with the other paired question set of "I imagine people will learn to use this system quickly"/"I think I would need help from someone with technical knowledge to use the system," performed the best, with 100% positive results consisting of strongly agree and agree for the question "Learn to use it quickly." However, the question "I think the system presents many inconsistencies" had a result of 18%; therefore, at least seven (43%) professionals marked it as neutral, agree, or strongly agree.

### Behavioral change results

A Likert scale was applied for questions related to confidence, ranging between 1–5, where 1 indicates "Strongly Disagree" and 5 indicates "Strongly Agree." For the confidence questions, the average results were: "With the system, I felt more confident in choosing antibiotics" (5), "With the system, I felt more confident in deciding the treatment duration" (3), and "I do not trust the response given by the system" (1). Scores related to choosing antibiotics and deciding treatment had average values corresponding to "Agree" and "Strongly Agree." However, for not trusting the response given by the chatbot, we believe that this value was higher than expected. This is due to the inability of the bot to retrieve all protocols that were trained or cases of greater complexity.

### Information retrieval

A test comprising 10 cases was created to evaluate the tool by the 20 selected physicians; some physicians did not answer all the questions, resulting in a total of 120 responses. In terms of the ability to retrieve information from the protocol, the bot retrieved the protocol in 83% of cases (100 instances), with 71% of these considered correct.

## DISCUSSION

The hypothesis that the tool demonstrates usability in terms of effectiveness, efficiency, and overall ease of use was validated by the overall score obtained, which classified the tool as good/excellent. In terms of ease of use, the chatbot was considered accessible, fast, and capable of being operated by only one person, in this case, a healthcare professional. The same applies to confidence in using this type of technology.

Overall, these results demonstrate the importance of usability in reducing the learning curve and enhancing the overall intuitiveness of the system. Such outcomes demonstrate the potential of usability to improve ease of accessibility, which is a critical feature for healthcare services and broader clinical environments. This finding aligns with the results reported by Grassini et al,^(19)^ who emphasized the central role of user experience in developing chatbots. Moreover, ease of use contributes to the intuitiveness and practicality of clinical decision support systems for healthcare professionals.^(20)^ Thus, this underscores the importance of working with medical professionals to design systems that are appropriate for healthcare needs, a conclusion supported by the results of the current study.

Regarding trust levels, the value for "not trusting the response" was higher than expected. This is due to the inability of the bot to retrieve all protocols that were trained or complex cases. To address this, future studies should focus on fine-tuning RAG, which could address the shortcomings in this version of the tool. This technical aspect directly influences the fact that, despite the positive outcomes concerning its usability and utility, a certain level of skepticism concerning the adoption of AI-based systems remains.

Healthcare professionals hesitate in adopting AI systems, which is often due to the potential risks associated with its use.^(21)^ This aspect is reflected in the current study, where potential errors related to hallucinated content and inaccurate responses were identified. These outcomes reinforce the findings from previous research by Laymouna et al.^(22)^ which highlights the importance of focusing on performance, usability, as well as addressing the ethical considerations surrounding the use of such systems, along with providing transparency in relation to potential limitations in its responses.

In terms of information retrieval, the discrepancy between the protocol retrieval and antibiotic accuracy is because of the format used for the input of the questions. In particular, the main problem identified for the antibiotic recommendation was the inability to retrieve the correct protocol. Refining the RAG pipeline and implementing granular retrieval strategies will be crucial in the future.

In addition to usability and trust outcomes, this study contributes to advancements in the field of antimicrobial stewardship, which remains an underexplored area in AI-based applications. As highlighted by Pinto et al.^(23)^ machine learning-driven tools, such as the one presented in this work, hold considerable promise and should be further explored, particularly for application in supporting clinical decision-making. The chatbot's potential lies not only in providing a rapid and efficient means of information retrieval but also in promoting adherence to institutional guidelines by recommending standardized treatments, which is recognized as an important factor in antimicrobial stewardship programs, as underscored by Krishnamoorthy.^(24)^

### Potential use cases

The tool demonstrated good usability, with a SUS score of 82, and effective retrieval of institutional antimicrobial protocols, suggesting its potential for integration into routine antimicrobial stewardship workflows. In practice, this system may assist clinicians in selecting appropriate therapy, confirming treatment duration, and rapidly accessing hospital guidelines at the point of care. Although further refinement is needed, the results obtained with this prototype highlight that LLM-based systems can provide efficient information retrieval, shorten the time required to reach guideline-concordant decisions, and support standardized antimicrobial use.

This study proposes integrating RAG methodology with an LLM to enhance antimicrobial treatment protocols across hospital departments. Implementing this methodology in the *Hospital Israelita Albert Einstein* protocols refined treatment specificity and accuracy. The system's capacity to generate responses from external data without extensive training is pivotal in ensuring alignment with hospital guidelines, enhancing diagnostic precision and treatment suitability, as quantitatively measured by a Jaccard index score and an SUS score of 82.

Validation using real-world applications, involving an assessment of 10 medical cases by specialists, confirms the utility and integration of the system into clinical workflows. The achieved SUS score of 82 indicates effective user interaction, supporting seamless adoption into daily practices. Moreover, the proficiency of the system in achieving an 83% success rate in protocol retrieval illustrates the operational advantage of the RAG methodology in complex clinical environments, potentially mitigating the risks associated with delayed or inaccurate treatment decisions.

## CONCLUSION

Future research should focus on exploring diverse retrieval methodologies to amplify the precision and responsiveness of medical information systems. Enhancing data types and incorporating real-time user feedback could optimize system performance. Furthermore, broadening the application to encompass various medical conditions and treatments could extend the benefits of advanced retrieval systems, potentially transforming medical practice in intensive care and other critical settings.

## Data Availability

The Likert scale chart and Python code generator are available at https://github.com/munai-health/sup-material-paper-BMchatbotrag. Additionally, this repository includes the prompt used for the chatbot.
